# Tumor growth rate as a metric of progression, response, and prognosis in pancreatic and intestinal neuroendocrine tumors

**DOI:** 10.1186/s12885-018-5257-x

**Published:** 2019-01-14

**Authors:** Clarisse Dromain, Marianne E. Pavel, Philippe Ruszniewski, Alison Langley, Christine Massien, Eric Baudin, Martyn E. Caplin, on behalf of the CLARINET Study Group, Austria M. Raderer, Austria M. Raderer, Belgium I. Borbath, D. Ysebaert, E. Sedláčková, P. Vítek, Denmark H. Grønbæk, France A. Adenis, L. Buscail, G. Cadiot, S. Dominguez, M. Ducreux, C. Lombard-Bohas, E. Mitry, P. Ruszniewski, J. F. Seitz, N. Begum, I. Harsch, M. Pavel, C. Schöfl, M. Weber, B. Wiedenmann, M. Mallath, P. Patil, K. Sambasivaiah, R. Saxena, E. Bajetta, A. Buonadonna, R. Buzzoni, R. Cannizzaro, A. Colao, C. De Angelis, P. Tomassetti, J. Ćwikła, B. Kos-Kudła, Slovakia T. Salek, J. Capdevila, G. Soler, J. M. Tabernero, H. Ahlman, M. Kjellman, G. Aithal, A. Anthoney, M. Caplin, A. Grossman, J. Newell-Price, J. Ramage, N. Reed, A. Rees, W. Steward, L. Wall, M. Choti, A. T. Phan, E. M. Wolin

**Affiliations:** 1 Department of Diagnostic and Interventional Radiology, CHUV University Hospital, Lausanne, Switzerland; 2 Department of Medicine 1, Division of Endocrinology and Diabetology, Friedrich-Alexander Universität Erlangen-Nürnberg, Universitätsklinikum Erlangen, Erlangen, Germany; 3 Division of Gastroenterology and Pancreatology, Beaujon Hospital, Clichy, France; 4 Faculty of Medicine, Paris Diderot University, Paris, France; 5 Ipsen, Boulogne-Billancourt, France; 6 APHP, Hypertension unit, Georges Pompidou European Hospital, F-75015 Paris, France; 7 Endocrine Tumour and Nuclear Medicine Unit, Gustave-Roussy Cancer Campus, Villejuif, France; 8 Neuroendocrine Tumour Unit, Department of Gastroenterology, Royal Free Hospital, London, UK

**Keywords:** Lanreotide, Neuroendocrine tumor, Prognostic factor, RECIST, Tumor growth rate

## Abstract

**Background:**

Lanreotide depot/autogel antitumor activity in intestinal/pancreatic neuroendocrine tumors (NETs) was demonstrated in the phase-3 CLARINET study (NCT00353496), based on significantly prolonged progression-free survival (PFS) versus placebo.

**Methods:**

During CLARINET, patients with metastatic intestinal/pancreatic NETs received lanreotide depot/autogel 120 mg or placebo every 4 weeks for 96 weeks. Imaging data (response evaluation criteria in solid tumors [RECIST] v1.0, centrally reviewed) were re-evaluated in this post hoc analysis of tumor growth rate (TGR) in NETs. TGR (%/month) was calculated from two imaging scans during relevant periods: pre-treatment (TGR_0_); 12–24 weeks before randomization versus baseline; each treatment visit versus baseline (TGR_Tx-0_); between consecutive treatment visits (TGR_Tx-Tx_). To assess TGR as a measure of prognosis, PFS was compared for TGR_0_ subgroups stratified by optimum TGR_0_ cut-off; a multivariate analysis was conducted to identify prognostic factors for PFS.

**Results:**

TGR_0_ revealed tumors growing during pre-treatment (median [interquartile range] TGR_0_: lanreotide 2.1%/month [0.2; 6.1]; placebo 2.7%/month [0.15; 6.8]), contrary to RECIST status. TGR was significantly reduced by 12 weeks with lanreotide versus placebo (difference in least-square mean TGR_0–12_ of − 2.9 [− 5.1, − 0.8], *p* = 0.008), a difference that was maintained at most subsequent visits. TGR_0_ > 4%/month had greater risk of progression/death than ≤4%/month (hazard ratio 4.1; [95% CI 2.5–6.5]; *p* < 0.001); multivariate analysis revealed lanreotide treatment, progression at baseline, TGR_0_, hepatic tumor load, and primary tumor type were independently associated with PFS.

**Conclusions:**

TGR provides valuable information on tumor activity and prognosis in patients with metastatic intestinal/pancreatic NETs, and identifies early lanreotide depot/autogel antitumor activity.

**Trial registration:**

Retrospective registration, 18 July 2006; EudraCT: 2005–004904-35; ClinicalTrials.gov: NCT00353496.

**Electronic supplementary material:**

The online version of this article (10.1186/s12885-018-5257-x) contains supplementary material, which is available to authorized users.

## Background

Neuroendocrine tumors (NETs) are slow-growing neoplasms that arise from diverse locations, including the pancreas and gastrointestinal (GI) tract. The prognosis of NETs is heterogeneous and tumor progression has been utilized in most phase-3 clinical trials to refine prognostic stratification. Current knowledge of well-differentiated NET growth kinetics and the relationship to treatment are limited; further understanding may improve the assessment of NET progression, prognosis and ultimately treatment.

Response evaluation criteria in solid tumors (RECIST) is used by oncologists and radiologists, and recognized by regulatory bodies as an assessment of tumor response to therapy, including NETs [1, 2]. RECIST estimates change in tumor burden using the sum of the longest diameters (SLD) of target lesions over a course of treatment which, together with the appearance of new lesions, is transformed into a categorical variable (complete response [CR], partial response [PR], stable disease [SD], or progressive disease [PD]) [1, 2].

Although a valuable tool widely used in clinical trials, RECIST is a qualitative variable, overemphasizing tumor shrinkage as a successful response to treatment, and requires improved reproducibility [3, 4]. Limitations of RECIST extend to accurate assessment of NET disease status, which can be confounded by their slow growth kinetics [5]. As a result, it can be difficult for practitioners to decide how to adapt treatment. Therefore, a metric that provides a more sensitive measure of tumor growth would prove advantageous in slow-growing tumor assessment, including NETs.

An assessment providing dynamic and quantitative evaluation of tumor kinetics may offer a useful complement to RECIST. Tumor slope has been explored as a prognostic factor in studies on solid tumors [6–10], including well-differentiated NETs [9, 10]. Tumor growth rate (TGR), which is based on change in tumor volume, has been independently associated with progression-free survival (PFS) using data from phase-1 studies in solid tumors (including upper GI and pancreatic tumors) [11], and with PFS and overall survival (OS) using data from the phase-3 TARGET study (sorafenib compared with placebo) in metastatic renal cell carcinoma (mRCC) [12]. More recently, an association of TGR with PFS has been reported in a post hoc analysis from a phase-2 single-arm trial of lanreotide depot/autogel 120 mg for non-functioning intestinal/pancreatic NETs in Japanese patients [13].

The CLARINET core study demonstrated the antitumor efficacy of lanreotide depot/autogel 120 mg/4 weeks in patients with non-functioning intestinal/pancreatic NETs compared with placebo [14]. The CLARINET open-label extension confirmed long-term safety and efficacy [15]. Most participants in the core study had SD at baseline according to RECIST v1.0. In these post hoc analyses, core study tumor growth measurements were evaluated using TGR as a measure of tumor progression and response before and during treatment, and as a prognostic factor for PFS.

## Methods

### Patients and study design

The design and methods for the CLARINET study have been described previously [14]. In brief, patients had unresectable, locally advanced or metastatic, well/moderately differentiated, somatostatin receptor-positive NETs with Ki-67 up to 10%. NETs were non-functioning except for gastrinomas controlled by proton-pump inhibitors for ≥4 months. Tumors originated in the pancreas, midgut or hindgut, or were of unknown origin.

The CLARINET study was an international, randomized, double-blind, placebo-controlled phase-3 trial (EudraCT: 2005–004904-35; ClinicalTrials.gov: NCT00353496). Patients received lanreotide 120 mg (*n =* 101) or placebo (*n =* 103) once every 4 weeks, for 96 weeks or until PD (assessed centrally using RECIST v1.0) or death. The study was conducted in accordance with the Declaration of Helsinki, Good Clinical Practice Guidelines, and local regulatory requirements. Trial documentation was approved by institutional review boards at each study site, and all patients provided written informed consent. A full list of the ethics committees and their institutions is provided Appendix 1 of in the Additional file 1.

### Assessments and endpoints

Study visits were scheduled during the screening period and at weeks 1 (baseline), 12, 24, 36, 48, 72, and 96 [14]. Baseline disease-progression status according to RECIST v1.0 was determined over a 12- to 24-week screening period [14]. Computed tomography or magnetic resonance imaging of the chest, abdomen and pelvis was performed twice during screening. Changes in target-lesion sizes were assessed from the second imaging test (performed 12–24 weeks after the first scan), and randomization took place within the following 4 weeks. Single scans were obtained at post-baseline visits and reviewed centrally according to RECIST v1.0. If a patient was withdrawn from the study prematurely for reasons other than PD/death, further imaging was undertaken (unless already undertaken within the previous 4 weeks).

TGR was expressed as the percentage change in tumor volume over 1 month (%/month): TGR = 100 × (exp(TG)–1), where TG = 3 × log(D2/D1)/time (months) [11, 12]. Tumor size (D) was determined using the SLD of target lesions only (according to RECIST v1.0); non-target and new lesions were not considered. D1 and D2 represent tumor sizes at evaluation dates 1 and 2; and time (months) = (date 2 – date 1 + 1)/30.44.

The clinical utility of TGR was assessed: by evaluating pre-treatment TGR (TGR_0_) using the two scans performed during screening; by calculating the change in TGR between pre-treatment and a visit during treatment (TGR_Tx–0_); and by calculating the TGR between consecutive visits during treatment (TGR_Tx–Tx_). To investigate the value of TGR_0_ as a prognostic factor for tumor progression (according to RECIST v1.0), the optimum TGR_0_ cut-off value (%/month) was determined, and PFS was compared for TGR_0_ subgroups stratified by the optimum TGR_0_ cut-off. Exploratory analyses were also conducted to identify potential prognostic factors for PFS (according to RECIST v1.0). Analyses were carried out to determine whether Ki-67 correlated with TGR_0_ in all patients with a tumor biopsy; and in a subgroup of patients with a tumor biopsy taken within 1 year of starting treatment.

### Statistical methods

A priori summary statistics were prepared for baseline characteristics based on the intention-to-treat (ITT) population (all randomized patients) [14]. All other analyses were post hoc and based on the ITT population, or a specified subset thereof. Spearman’s rank correlation was used to test for an association between Ki-67 at screening and TGR_0_. TGRs during the study were analyzed using mixed-model regression analyses with repeated measures; least square (LS) means and 95% confidence intervals (CIs) were calculated by visit for each treatment.

To explore TGR as a prognostic factor, a receiver operating characteristic (ROC) analysis was conducted to determine the optimum TGR_0_ cut-off value associated with risk of PD (according to RECIST v1.0) or death. PFS was estimated using the Kaplan–Meier method and compared between lanreotide and placebo according to subgroups defined by the TGR_0_ cut-off value using the log-rank test. Comparisons were also made between the TGR_0_ subgroups within each treatment group. In an additional analysis, PFS was estimated between lanreotide and placebo for the subgroups TGR_0_ > 4%/month and ≤ 10%/month; and TGR_0_ > 10%/month. To explore the prognostic value of absolute TGR at 12 weeks (TGR_0–12_), an association between TGR_0–12_ and PFS was assessed for lanreotide and placebo groups. Hazard ratios (HRs) were estimated from Cox proportional hazard models. Patients with PD due to non-target or new lesions were excluded from these subgroup analyses. The prognostic value of TGR_0_ was further explored using a Cox proportional hazards model to identify potentially important covariates (Wald Chi-square *p <* 0.10, Additional file 1: Table S1). These covariates were analyzed via a multivariate Cox proportional hazard model of PFS; only covariates potentially important in the presence of other terms (*p <* 0.10) remained in the final model. All statistical analyses were carried out using SAS statistical software (SAS V9.4).

## Results

### Patients

The patient population has been described previously [14]. Briefly, 204 patients were randomized (ITT population) to receive lanreotide (*n =* 101) or placebo (*n =* 103). Most patients had received no previous treatment (84%, 172/204) and had SD over the 12- to 24-week screening period before study entry (96%, 195/204), according to RECIST v1.0 [14]. Mean (standard deviation) time between diagnosis and enrollment was 33.5 (43.7) months. All patients had grade 1 or low-grade 2 tumors (Ki-67 < 10%). Overall, 33% (67/204) of patients had hepatic tumor loads over 25%; 45% (91/204) had primary tumors of the pancreas and 36% (73/204) of the midgut. In total, 200 patients with evaluable data and available SLD measurements were included in these post hoc analyses. Patients were excluded if they had: missing SLD; a change in TGR that could not be measured; or non-target or new lesions (Additional file 1: Figure S1).

### Use of TGR to measure tumor progression and response before and during treatment

**Fig. 1:**
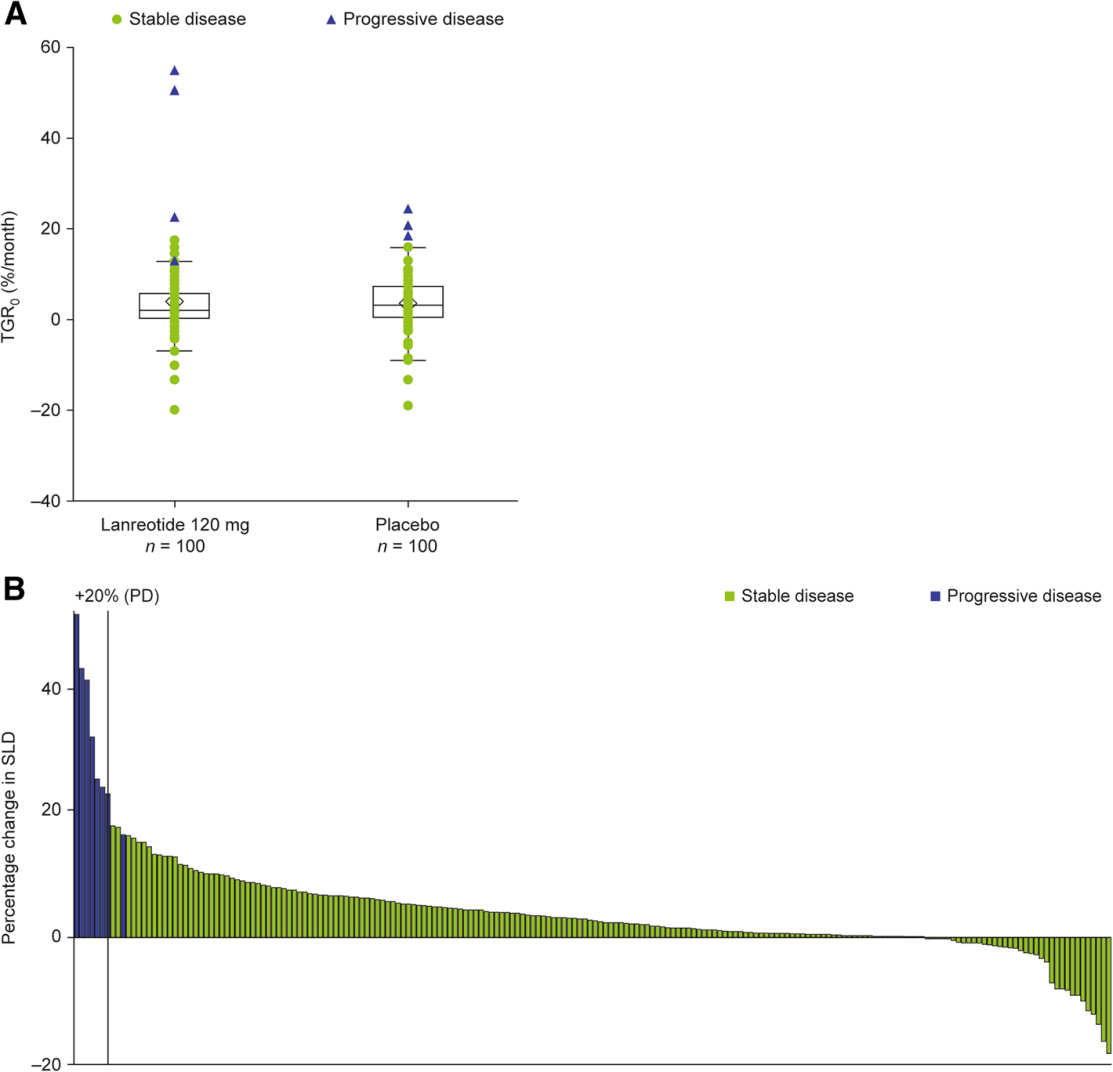
Distribution of (**a**) TGR_0_ and (**b**) percentage change in SLD among patients during the pre-treatment period. **a** Distribution of TGR0 among patients during the pre-treatment period according to RECIST v1.0 classification. Individual data points represent the TGR0 in individual patients (ITT population), according to their classification by RECIST v1.0. Boxes represent median and interquartile range (Q1–Q3), whiskers represent the minimum observation still within 1.5 IQR of lower quartile (Q1), and the maximum observation still within 1.5 IQR of the upper quartile (Q3). Median (IQR) TGR0: 2.1%/month (0.2; 6.1) (lanreotide 120 mg); 2.7%/month (0.15; 6.8) (placebo). **b** Percentage changes in SLD during the pre-treatment period among individual patients (ITT population). Data are sorted from high percentage change in SLD to low percentage change in SLD over the pre-treatment period. A patient with a change in SLD less than 20% had PD due to the presence of new lesions. Lanreotide 120 mg, n = 100; placebo, n = 100. ITT, intention-to-treat; IQR; interquartile range; PD, progressive disease; RECIST, response evaluation criteria in solid tumors; SLD, sum of longest diameters; TGR0, pre-treatment tumor growth rate

The distributions of patient TGR_0_ during the pre-treatment period were similar for both treatment groups (post hoc analyses; ITT population; Fig. 1a). Overall, patients receiving lanreotide had a median (interquartile range) TGR_0_ of 2.1%/month (0.2–6.1), while those receiving placebo had 2.7%/month (0.15–6.8). The majority of patients, including those with SD, had increases in SLD measurements during the pre-treatment period (Fig. 1b). We observed no correlation between Ki-67 at screening and TGR_0_ in analyses that included either all patients with a tumor biopsy, or a subgroup of patients with a tumor biopsy taken within 1 year of starting treatment (Additional file 1: Figure S2).

**Fig. 2:**
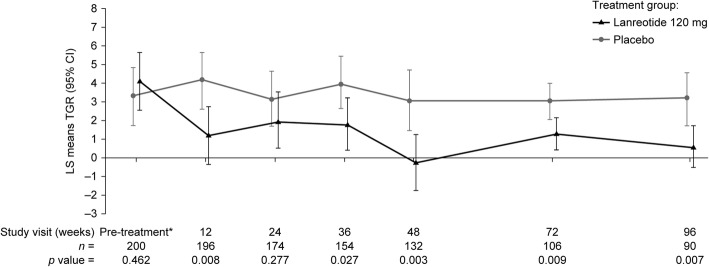
Estimated TGR_0_ and TGR_Tx–Tx_. *Pre-treatment TGR (TGR_0_) is calculated from the second imaging test during the screening period (performed 12–24 weeks after the first screening period scan). LS means and *P*-values are derived from a mixed model with repeated measures. CI, confidence interval; LS, least squares; TGR, tumor growth rate; TGR_0_, pre-treatment TGR, TGR_Tx–Tx_, TGR between consecutive study visits during treatment

When using TGR to measure proliferative activity during treatment, variation between TGR_0_ and each study visit (TGR_Tx–0_) according to RECIST v1.0 status revealed that a large proportion of patients had reductions in TGR at post-treatment visits relative to TGR_0_ (Additional file 1: Figure S3), with greater reductions in lanreotide compared with the placebo group. These findings were despite most patients being classified as SD with RECIST v1.0, regardless of treatment. A disparity between median TGR_0_ and LS mean TGR_0_ in the lanreotide group was due to high TGR_0_ in two patients. Calculation of TGRs between consecutive visits during treatment (TGR_Tx–Tx_) revealed a reduction in TGR as early as week 12 of lanreotide treatment (TGR_0–12_), resulting in a statistically significant difference between treatment groups that was maintained throughout most of the treatment period (Fig. 2; Additional file 1: Table S2). Individual TGRs (TGR_Tx–Tx_) for patients with PD after the start of treatment revealed that, in many cases, PD was a result of accumulated tumor growth over time, rather than a sudden increase in TGR (Additional file 1: Figure S4).

### Use of TGR_0_ as a prognostic factor

**Fig. 3:**
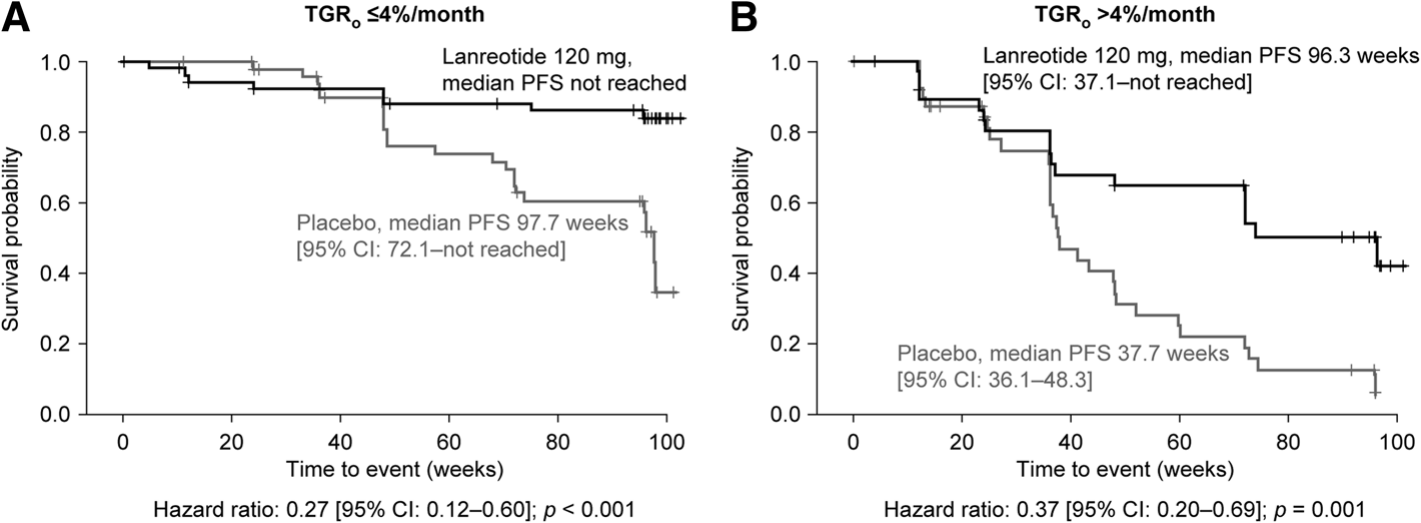
PFS in patients with (**a**) TGR_0_ ≤ 4%/month and (**b**) TGR_0_ > 4%/month. Analysis of PFS considers centrally assessed disease progressions (using RECIST v1.0 criteria) and any deaths reported during the study as events. A total of 14 patients in progression purely due to non-target or new lesions were excluded. TGR_0_ ≤ 4%, lanreotide *n =* 56; placebo *n =* 53; TGR_0_ > 4%, lanreotide *n =* 38; placebo *n =* 40. HRs are derived from a Cox proportional hazards model. *P*-values are derived from the log-rank test. CI, confidence interval; HR, hazard ratio; PFS, progression-free survival; TGR, tumor growth rate; TGR_0_, pre-treatment TGR

Of the TGR_0_ thresholds assessed by ROC analysis, a cut-off TGR_0_ of 4%/month during the pre-treatment period had optimal association with the risk of PD (according to RECIST v1.0 status) or death independently of treatment group (Additional file 1: Figure S5). When comparing PFS between TGR_0_ subgroups, TGR_0_ > 4%/month was associated with a four-fold greater risk of PD/death than TGR_0_ ≤ 4%/month in the overall population (HR 4.1 [95% CI [2.5–6.5]; *p <* 0.001, *n =* 187), and within each treatment group (Additional file 1: Figure S6). When considering PFS within TGR_0_ subgroups between treatment groups, lanreotide was significantly more effective than placebo at reducing the risk of PD/death, by 73 and 63% in those with TGR_0_ ≤ 4%/month and > 4%/month, respectively (Fig. 3). In a separate analysis, lanreotide reduced the risk of PD/death by 73% compared with placebo in a subgroup of patients with TGR_0_ > 4%/month and ≤ 10%/month (Additional file 1: Figure S7). There was a trend towards increased PFS with lanreotide compared with placebo in patients with TGR_0_ > 10%/month, however this did not reach statistical significance. Thirteen patients considered to show disease progression based only on non-target or new lesions were excluded from these analyses (lanreotide, *n =* 6; placebo, *n =* 7). The prognostic value of early on-treatment TGR (TGR_0–12_) was also explored. Analyses within each treatment group indicated that a higher TGR_0–12_ was associated with worse PFS in both lanreotide (HR for a 10%/month increase in TGR_0–12_: 8.0 [95% CI [3.0; 21.3], *p* < 0.0001, *N* = 90) and placebo groups (HR for a 10%/month increase in TGR_0–12_: 8.8 [95% CI [4.4; 17.6], *p* < 0.0001, *N* = 93).

**Fig. 4:**
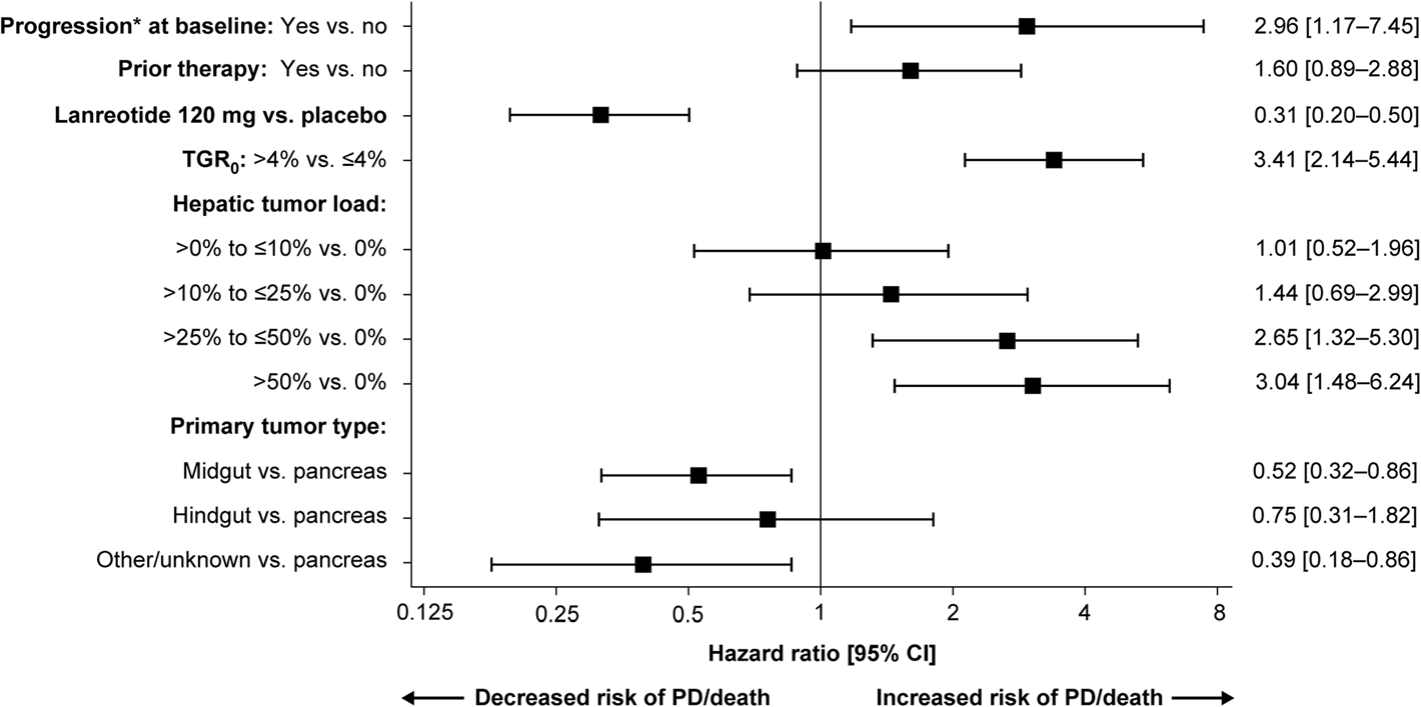
Potential prognostic factors for PFS (final multivariate model). *According to RECIST v1.0. Hazard ratios [95% CI] for centrally assessed PD or death were generated from a post hoc multivariate Cox proportional hazards model. Four patients with missing TGR_0_ were excluded from the model. There were limited patients with progression at baseline (*n =* 8) and primary tumor type: hindgut *n =* 14. CI, confidence interval; PD, progressive disease; PFS, progression-free survival; RECIST, response evaluation criteria in solid tumors; TGR_0_, pre-treatment tumor growth rate

An exploratory analysis of 15 potential prognostic factors for PFS identified seven potentially important covariates (*p <* 0.10; Additional file 1: Table S1). A separate analysis, restricted to the subgroup of patients with tumor biopsies taken within 1 year of treatment start, confirmed that neither Ki-67 (*p* = 0.5917; *N* = 126) nor tumor grade (*p* = 0.5294; *N* = 125) were potentially important prognostic factors for PFS in this subgroup of patients. When the seven potentially important covariates were included into a multivariate model, chromogranin A (CgA) levels at baseline, body mass index (BMI), sex and tumor grade were no longer significant at *p* = 0.10 in the presence of the other terms and were, therefore, excluded from the final multivariate model. The final model showed TGR_0_ > 4%, hepatic tumor load (> 25%–≤50 and > 50%), progression at baseline, and a pancreatic primary tumor were independent prognostic factors for worse PFS (according to RECIST v1.0), while prior therapy was not prognostic for PFS in the presence of other terms (Fig. 4). After adjustment for these prognostic factors, lanreotide reduced the risk of progression or death by 69% versus placebo. Risk of PD/death was more than three-fold higher in patients with TGR_0_ > 4%/month.

## Discussion

These post hoc analyses were conducted to evaluate the clinical utility of TGR as a measure of tumor progression, response and prognosis in patients with intestinal/pancreatic NETs receiving lanreotide or placebo, using data from the CLARINET study [14]. Analyses of the use of TGR to assess tumor progression before treatment (TGR_0_) revealed a large proportion of patients’ tumors were actively growing during the pre-treatment period, despite classification as SD according to RECIST v1.0. There was no correlation between Ki-67 at screening and TGR_0_, despite an attempt to stratify the data by only including patients with biopsies taken within 1 year of treatment start. Analyses of the use of TGR to assess proliferative activity during treatment (TGR_Tx–0_) showed a large proportion of patients had reductions in TGR_Tx–0_, which tended to be greater with lanreotide compared with placebo, while most patients were still classified as SD according to RECIST v1.0. TGR_Tx–Tx_ demonstrated the antitumor efficacy of lanreotide compared with placebo, as early as 12 weeks into treatment, by reduction and subsequent stabilization. Analyses of the use of TGR_0_ as a prognostic factor showed that by using a TGR_0_ cut-off of 4%/month (found to have optimal association with risk of PD/death by ROC analysis), patients with TGR_0_ > 4%/month had a four-fold higher risk of PD/death compared with ≤4%/month, in the overall population and within both treatment groups. TGR_0–12_ was also found to be prognostic for PFS in both treatment groups. A multivariate analysis identified five factors independently associated with PFS: lanreotide treatment, progression at baseline, TGR_0_, hepatic tumor load (> 25%–≤50 and > 50%), and primary tumor type. Ki-67 at baseline was not identified as a potentially important prognostic factor for PFS, prior therapy (yes) was not prognostic in the presence of other terms and, notably, CgA at baseline, and tumor grade were excluded from the final model.

The findings from the present analyses accord with other similar analyses. The advantage of TGR as a rapid measure of tumor response has been reported previously; TGR identified antitumor efficacy during treatment at the first tumor evaluation in phase-1 and -3 studies of mRCC [11, 12, 16]. The utility of TGR in non-functioning intestinal/pancreatic NETs was first described in a recent single-arm phase-2 trial of 32 Japanese patients [13]. A numerical reduction in TGR was identified within 12 weeks of initiating lanreotide treatment, despite 65% of patients being classified as PD at baseline (RECIST v1.1), and was sustained from pre-treatment to last-value [13]. Our analyses expand upon this study in four ways: firstly, they reveal that TGR can detect early differences between lanreotide and placebo groups, despite the slow growth kinetics of NETs; secondly, they demonstrate sustained lanreotide antitumor activity compared with placebo using the large dataset from CLARINET; thirdly they provide additional evidence that although lanreotide reduced TGR versus placebo overall, tumor growth was ongoing in many patients, including those classified as having SD; and finally, in many cases a PD event was the result of ongoing tumor growth, rather than the result of a sudden increase in TGR. Our findings regarding the utility of TGR during treatment (TGR_Tx_) for monitoring tumor response and the durability of drug effect also resonate with other analyses from phase-2 and -3 trials in other tumor types [6, 12, 17, 18]. TGR_0_ measurements were suggestive of actively growing tumors and, as seen with other tumor types, TGR_0_ did not reflect RECIST status during pre-treatment and early treatment evaluation [11, 12, 19]. Others have highlighted the inadequacies of RECIST as a measure of tumor response because it involves condensing information into four categories that are defined before treatment, regardless of growth kinetics. Additionally, RECIST may not always be relevant for slow-growing tumors or treatments that stabilize growth, and any observed pseudoprogression, even if uncommon in NETs, may be misrepresented as PD by RECIST [3, 19, 20]. Our findings that a TGR_0_ cut-off of 4%/month and TGR_0–12_ are prognostic for PFS are consistent with others who identified an association between growth kinetics and clinical outcomes. In a large retrospective analysis of 20 phase-1 mRCC trials, a 9% decrease in progression hazard was observed with every 10% decrease in TGR (reference compared with experimental period) [11], and TGR was associated with PFS regardless of treatment (sorafenib or placebo) in a retrospective analysis from the phase-3 TARGET trial in mRCC [12]. A new response metric for glioblastoma incorporating a linear model of radial tumor expansion computed at first post-radiation scan was prognostic for PFS, and agreed with more complex anatomic and spherical equivalents [21, 22]. In addition, response outcomes utilizing growth dynamics have also been previously shown to be prognostic for OS in renal and prostate cancer [12, 17, 18, 21–23]. The TGR_0_ cut-off of 4%/month could, therefore, be of value within routine clinical practice by providing a prognosis for PFS before treatment start, thus allowing clinicians to make earlier decisions regarding future treatment. TGR_Tx-0_ would also be potentially useful in the clinic to identify patients who are or are not benefitting early in the course of their treatment (e.g. TGR_12–0_). TGR_0_ (≤/> 4%/month), if confirmed to be prognostic for other therapies, and TGR_Tx-0_ would be of particular importance when planning and monitoring more toxic treatments, such as peptide receptor radionuclide therapy and chemotherapy, ensuring these are continued only for as long as is necessary.

The lack of correlation between Ki-67 and TGR_0_ in this analysis was surprising. However, known difficulties in assessment of the Ki-67 index (intra- and intertumoral staining heterogeneity and counting methods, for example) may, in part, account for this [24, 25]. Ki-67 was not identified as a potentially important prognostic factor for PFS from the exploratory multivariate analysis, despite the known importance of Ki-67 as a prognostic marker in NETs [26]. Nevertheless, our findings accord with a previous exploratory analysis of prognostic factors using data from the CLARINET study [27]. Despite being of potential interest in the univariate setting, CgA levels at baseline, tumor grade, BMI, and sex were excluded from the final multivariate model presented here. Reassuringly, this resonates with previous analyses of prognostic factors for PFS [13, 27], suggesting CgA levels and tumor grade are less robust prognostic factors than hepatic tumor load and TGR_0_ in patients with NETs.

This study was not without limitations. There are inherent limitations in post hoc analyses that potentially limit their interpretation. Any confounding due to anisotropy was not accounted for, as target lesions were assumed to be spherical, although this does tend to be the case for liver metastases. Target lesions followed for TGR may be slow growing, and all lesions within a patient were assumed to be similar; therefore, TGR for an individual may not be representative of their overall tumor targets. Inaccuracies in TGR may be introduced by errors in SLD measurements and, as tumor growth was assumed to be exponential, deviations from this growth pattern; in addition, for TGR_Tx-0_, lesions used for the tumor assessments during the screening period did not have to be the same lesions as those assessed during the treatment period. In concordance with the SLD calculation used in RECIST v1.0, non-target and new lesions were not considered in TGR calculations. The Ki-67 and TGR_0_ correlation analysis and exploratory analysis of the prognostic value of Ki-67 were limited due to a number of reasons. Firstly, many tumor biopsies were collected several years before the start of treatment, although additional analyses (restricted to the subgroup in whom biopsies were taken in the year before treatment initiation) provided similar results to the overall population. Secondly, Ki-67 data were either unreliably quantified or missing for 41 patients who were enrolled into the CLARINET study based on mitotic index. Thirdly, a number of patients had Ki-67 values recorded as < 1% (*N* = 15) or < 2% (*N* = 29), which restricted the way Ki-67 data could be handled; and few patients (*N* = 17) with Ki-67 values > 5% were included. The ROC area-under-curve implied that TGR_0_ 4%/month cut-off was not strongly deterministic of PFS. Further validation will be required to determine whether this cut-off is relevant in other study populations. Thirteen patients with progression based on non-target or new lesions were excluded from the prognostic value analysis of the TGR_0_ 4%/month cut-off. However, as a similar number of patients were excluded in each treatment group, it is unlikely to have affected the results.

These limitations notwithstanding, our findings suggest TGR has potential clinical utility as a novel metric for proliferative activity, particularly in future studies of somatostatin analogs or novel targeted therapies in NETs in which subtle changes in tumor growth are expected but may not be identified using RECIST. Further advantages of TGR utility include its potential in individualizing patient treatment, with the possibility that therapy can be adjusted based on a more precise analysis of tumor kinetics.

## Conclusion

Overall, these post hoc analyses of TGR to assess activity before and during treatment revealed that TGR_0_ and TGR_Tx_ provided valuable information on tumor kinetics. Evaluation of TGR_0_ as a prognostic factor revealed TGR_0_ was prognostic for PFS, irrespective of treatment. Furthermore, lanreotide was more effective than placebo in delaying PD/death, irrespective of the TGR_0_ 4%/month cut-off. Future perspectives include the development of TGR-based categorical definitions of disease status, and further validation of TGR as a predefined endpoint in prospective studies.

## Additional file

Additional file 1: **Table S1.** Categories tested in the exploratory multivariate analyses of potential prognostic factors. **Table S2.** TGR0 and TGRTx–Tx for patients receiving lanreotide or placebo (%/month). **Figure S1.** Patient disposition in the CLARINET TGR analysis. **Figure S2.** Correlation between TGR0 and Ki-67 at screening in (a) all patients and (b) in a subgroup of patients with a tumor biopsy taken within 1 year of the start of treatment (ITT population). **Figure S3.** Variation in TGR and tumor response evaluation by RECIST v1.0 between pre-treatment and each treatment visit (TGRTx-0) at (a) Week 12; (b) Week 24; (c) Week 36; (d) Week 48; (e) Week 72; and (f) Week 96. **Figure S4.** Individual TGRs (TGRTx–Tx) for patients with PD after the start of treatment within (a) lanreotide and (b) placebo treatment groups. **Figure S5.** Determination of the optimum TGR0 cut-off value. **Figure S6.** PFS between TGR0 subgroups (≤4%/month and >4%/month) within (a) lanreotide and (b) placebo treatment groups. **Figure S7.** PFS between lanreotide and placebo groups within TGR0 subgroups (a) >4%/month and ≤10%/month and (b) >10%/month. Appendix 1: List of Ethics Committees and/or Institutional Review Boards. (DOCX 1446 kb)

## Abbreviations

BMI: Body mass index
CgA: Chromogranin A
CI: Confidence interval
CR: Complete response
GI: Gastrointestinal
HR: Hazard ratio
ITT: Intention-to-treat
LS: Least square
mRCC: Metastatic renal cell carcinoma
NET: Neuroendocrine tumor
OS: Overall survival
PD: Progressive disease
PFS: Progression-free survival
PR: Partial response
RECIST: Response evaluation criteria in solid tumors
ROC: Receiver operating characteristic
SD: Stable disease
SLD: Sum of the longest diameters
TGR: Tumor growth rate
