# Supplementary material for: Tumor growth rate as a metric of progression, response, and prognosis in pancreatic and intestinal neuroendocrine tumors
Source: BMC Cancer. 2019 Jan 14;19:66. doi: 10.1186/s12885-018-5257-x (PMC6332566; doi:10.1186/s12885-018-5257-x)
Supplement: Supplementary file 1 — Table S1. Categories tested in the exploratory multivariate analyses of potential prognostic factors. Table S2. TGR0 and TGRTx–Tx for patients receiving lanreotide or placebo (%/month). Figure S1. Patient disposition in the CLARINET TGR analysis. Figure S2. Correlation between TGR0 and Ki-67 at screening in (a) all patients and (b) in a subgroup of patients with a tumor biopsy taken within 1 year of the start of treatment (ITT population). Figure S3. Variation in TGR and tumor response evaluation by RECIST v1.0 between pre-treatment and each treatment visit (TGRTx-0) at (a) Week 12; (b) Week 24; (c) Week 36; (d) Week 48; (e) Week 72; and (f) Week 96. Figure S4. Individual TGRs (TGRTx–Tx) for patients with PD after the start of treatment within (a) lanreotide and (b) placebo treatment groups. Figure S5. Determination of the optimum TGR0 cut-off value. Figure S6. PFS between TGR0 subgroups (≤4%/month and >4%/month) within (a) lanreotide and (b) placebo treatment groups. Figure S7. PFS between lanreotide and placebo groups within TGR0 subgroups (a) >4%/month and ≤10%/month and (b) >10%/month. Appendix 1: List of Ethics Committees and/or Institutional Review Boards. (DOCX 1446 kb) [file 12885_2018_5257_MOESM1_ESM.docx]

**Supplementary Material**

**Tumor growth rate as a metric of progression, response, and prognosis in pancreatic and intestinal neuroendocrine tumors**

Clarisse Dromain MD PhD, Marianne E. Pavel MD, Philippe Ruszniewski MD PhD, Alison Langley MSc, Christine Massien MD, Eric Baudin, MD PhD, Martyn E. Caplin DM, on behalf of the CLARINET Study Group

**Table S1.** Categories tested in the exploratory multivariate analyses of potential prognostic factors.

| **Covariate** | **Categories** | ***P*-value** |
| --- | --- | --- |
| Age | ≤65 years (reference)  >65 years | 0.5105 |
| **BMI** | ≤Median value (reference)  >Median value | 0.0657 |
| **CgA at baseline** | ≤ULN  1–2 ULN  >2 ULN | 0.0111 |
| Ethnicity | Caucasian/White (reference)  Other | 0.5279 |
| **Tumor grade** | G1 (reference)  G2 | 0.0981 |
| **Hepatic tumor load** | 0% (reference)  >0% to ≤10%  >10% to ≤25%  >25% to ≤50%  >50% | <0.0001 |
| Ki-67 at baseline | ≤2% (reference)  >2% to ≤5%  >5% to <10%  >10% | 0.2402 |
| **Primary tumor type** | Pancreas (reference)  Midgut  Hindgut  Other/unknown | 0.0232 |
| Previous chemotherapy for NF-NET | Yes  No (reference) | 0.9648 |
| Previous surgery of the primary tumor | Yes (reference)  No | 0.9912 |
| Region | Western Europe  Eastern Europe and India  USA (reference) | 0.7712 |
| **Sex** | Male (reference)  Female | 0.0830 |
| **TGR_0_** | ≤4%/month  >4%/month | <0.0001 |
| Time since diagnosis | ≤5 years (reference)  >5 years | 0.7787 |
| USA/non-USA enrollment | USA  Non-USA (reference) | 0.8396 |

Covariates were tested individually in a Cox proportional hazards model, with treatment and the two baseline stratification factors: progression at baseline (yes, no) and previous therapy for NF-NET at entry (yes, no). Covariates with a Wald Chi-square *p*-value <0.10 were considered potentially important (indicated in bold and grey) and were entered into a multivariate Cox proportional hazards model. BMI, body mass index; CgA, chromogranin A; G, tumor grade; NF-NET, non-functioning neuroendocrine tumor; TGR_0_, pre-treatment tumor growth rate; ULN, upper limit of normal range.

**Supplementary Table 2.** TGR_0_ and TGR_Tx–Tx_ for patients receiving lanreotide or placebo (%/month).

| **Treatment period** | **TGR**  **measure** |  | **n** | **Lanreotide,  LS means (95% CI)** | **Placebo,  LS means (95% CI)** | **Treatment difference in**  **LS means (95% CI); *p*-value** |
| --- | --- | --- | --- | --- | --- | --- |
| Pre-treatment^a^ | TGR_0_ |  | 200 | 4.1 (2.6 to 5.6) | 3.3 (1.7 to 4.8) | 0.8 (–1.4 to 3.0); *p =* 0.46 |
| Week 0^b^–12 | TGR_0–12_ |  | 196 | 1.2 (–0.4­ to 2.7) | 4.1 (2.6 to 5.6) | –2.9 (–5.1 to –0.8); *p =* 0.008 |
| Week 12–24 | TGR_12–24_ |  | 174 | 2.0 (0.5 to 3.5) | 3.2 (1.7 to 4.6) | –1.2 (–3.3 to 0.9); *p =* 0.28 |
| Week 24–36 | TGR_24–36_ |  | 154 | 1.8 (0.4 to 3.2) | 4.0 (2.6 to 5.4) | –2.2 (–4.2 to –0.3); *p =* 0.03 |
| Week 36–48 | TGR_36–48_ |  | 132 | –0.3 (–1.8 to 1.2) | 3.1 (1.5 to 4.7) | –3.4 (–5.6 to –1.1); *p =* 0.003 |
| Week 48–72 | TGR_48–72_ |  | 108 | 1.3 (0.4 to 2.1) | 3.0 (2.0 to 4.0) | –1.7 (–3.0 to –0.4); *p =* 0.009 |
| Week 72–96 | TGR_72–96_ |  | 90 | 0.6 (–0.5 to 1.7) | 3.1 (1.7 to 4.6) | –2.6 (–4.4 to –0.7); *p =* 0.007 |

^a^Pre-treatment period is defined as the period between the first and second scans undertaken during the screening period; ^b^baseline scan at the end of screening period and start of treatment. CI, confidence interval; LS, least squares; TGR, tumor growth rate; TGR_0_, pre-treatment TGR; TGR_Tx–Tx_, TGR between consecutive study visits during treatment. LS means for TGR_0_ and TGR_Tx–Tx_ and *p*-values are derived from a mixed model with repeated measures.

**Supplementary Fig. 1.** Patient disposition in the CLARINET TGR analysis.

**

**

LAN, lanreotide depot; PBO, placebo, SLD, sum of longest diameters, TGR, tumor growth rate; TGR_0_, pre-treatment TGR; TGR_Tx–0_; change in TGR between pre-treatment and a visit during treatment; TGR_Tx–Tx_, TGR between consecutive visits during treatment.

**Supplementary Fig. 2.** Correlation between TGR_0_ and Ki-67 at screening in **(A)** all patients and **(B)** in a subgroup of patients with a tumor biopsy taken within 1 year of the start of treatment (ITT population).





Correlation analysis of TGR_0_ and Ki-67 at screening. **(A)** Rho = 0.106, *p =* 0.1849; *N =* 159 **(B)** Rho = 0.046, *p =* 0.6499; *N =* 99 (patients with Ki-67 that could not be reliably quantified, or missing TGR_0_ were excluded). Patients with Ki-67 recorded as ‘<1%’ or ‘<2%’ were analyzed as 1% and 2%, respectively. Patients with Ki-67% ‘<10%’ were set to missing. ITT, intention-to-treat; TGR_0_, pre-treatment tumor growth rate.

**Supplementary Fig. 3.** Variation in TGR and tumor response evaluation by RECIST v1.0 between pre-treatment and each treatment visit (TGR_Tx-0_) at **(A)** Week 12; **(B)** Week 24;
**(C)** Week 36; **(D)** Week 48; **(E)** Week 72; and **(F)** Week 96.

**
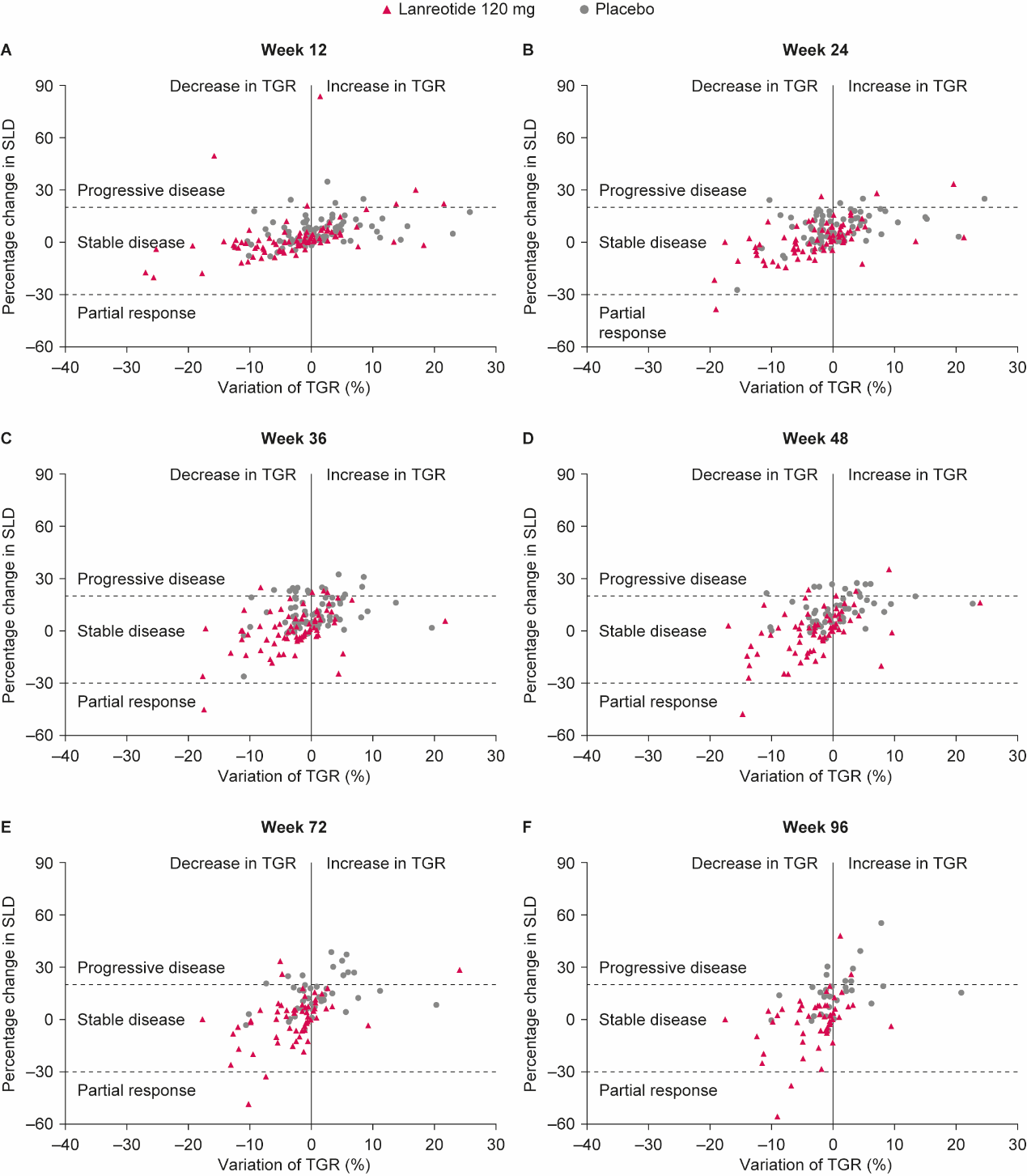
**

Variation of TGR (%, individual values) according to the tumor response by RECIST v1.0 (SLD % change), from pre-treatment to each treatment period (weeks 12, 24, 36, 48, 72, and 96). Patients were excluded if they had a missing SLD evaluation at the visit, or if the change in TGR could not be calculated. SLD, sum of longest diameters; TGR, tumor growth rate;
TGR_Tx–0_, change in TGR calculated from pre-treatment TGR and each treatment visit.

**Supplementary Fig. 4.** Individual TGRs (TGR_Tx–Tx_) for patients with PD after the start of treatment within **(A)** lanreotide and **(B)** placebo treatment groups.


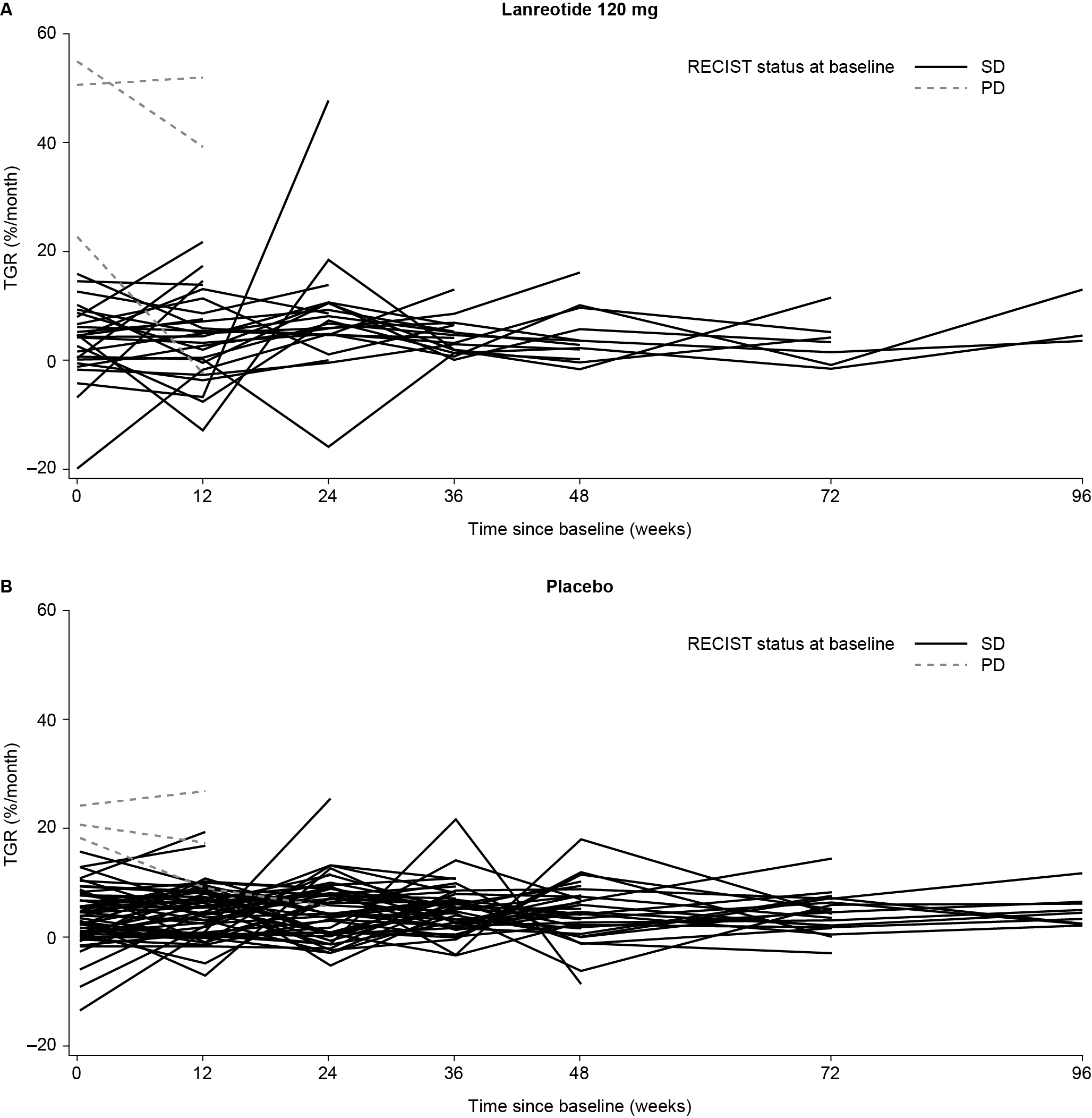


All patients with disease progression after the start of study treatment are shown, including patients with PD due to non-target or new lesions. Lanreotide 120 mg: *N* = 29; Placebo *N* = 58.

SD, stable disease; PD, progressive disease; RECIST, response evaluation criteria in solid tumors; TGR, tumor growth rate; TGR_Tx–Tx_: TGR between consecutive treatment visits.

**Supplementary Fig. 5.** Determination of the optimum TGR_0_ cut-off value.


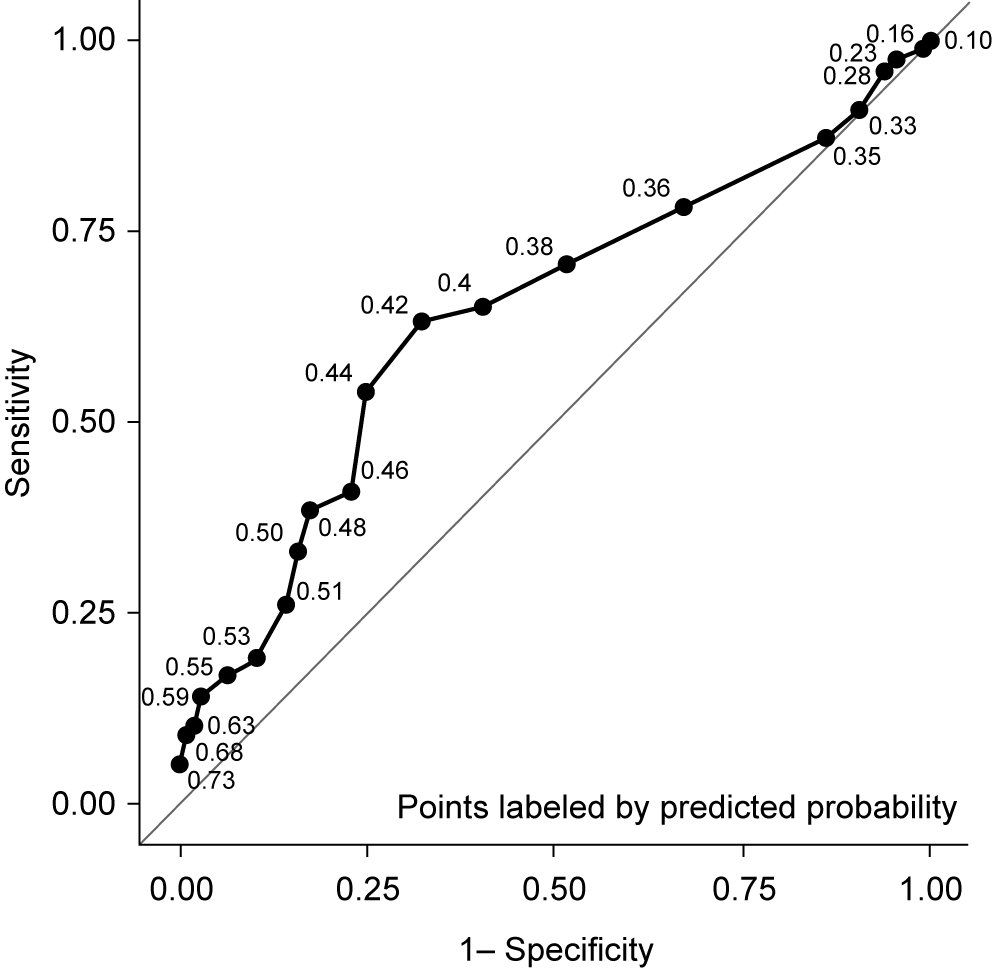


The point with the predictive probability of 0.42 (the closest data point to the top left corner of the ROC curve) is the value of most interest. The predictive probability of 0.42 corresponds with a TGR cut-off = 4%/month. The AUC of 0.6403 is a measure of accuracy of the result, where an AUC = 1.00 is perfect and an AUC = 0.5 is not reliable. A total of 14 patients considered to show disease progression purely due to non-target or new lesions were excluded. AUC, area under the ROC curve; ROC, receiver operating characteristic; TGR_0_, pre-treatment tumor growth rate.

**Supplementary Fig. 6:** PFS between TGR_0_ subgroups (≤4%/month and >4%/month) within **(A)** lanreotide and **(B)** placebo treatment groups.





This analysis of PFS time considers as events: centrally assessed disease progressions (using RECIST v1.0) and any deaths reported during the study. Patients with disease progression due to non-target or new lesions were excluded. Lanreotide depot 120 mg: TGR_0_ ≤4%/month *N =* 56, TGR_0_ >4%/month *N =* 38. Placebo: TGR_0_ ≤4%/month *N =* 53, TGR_0_ >4%/month *N =* 40. CI, confidence interval; PFS, progression-free survival; RECIST, response evaluation criteria in solid tumors; TGR_0_, pre-treatment tumor growth rate.

**Supplementary Fig. 7:** PFS between lanreotide and placebo groups within TGR_0_ subgroups **(A)** >4%/month and ≤10%/month and **(B)** >10%/month

**

**

This analysis of PFS time considers as events: centrally assessed disease progressions (using RECIST v1.0) and any deaths reported during the study. Patients with disease progression due to non-target or new lesions were excluded. TGR_0_ >4%/month and ≤10%/month: lanreotide depot 120 mg: *N* = 27; placebo: *N* = 26. TGR_0_ >10%/month: lanreotide depot 120 mg: *N* = 11; placebo: *N* = 14.CI, confidence interval; PFS, progression-free survival; RECIST, response evaluation criteria in solid tumors; TGR_0_, pre-treatment tumor growth rate.

**Appendix 1**

**CLARINET Study Group**

**Austria** M. Raderer; **Belgium** I. Borbath, D. Ysebaert; **Czech Republic** E. Sedláčková, P. Vítek; **Denmark** H. Grønbæk; **France** A. Adenis, L. Buscail, G. Cadiot, S. Dominguez, M. Ducreux, C. Lombard-Bohas, E. Mitry, P. Ruszniewski, J.F. Seitz; **Germany** N. Begum, I. Harsch, M. Pavel, C. Schöfl, M. Weber, B. Wiedenmann; **India** M. Mallath, P. Patil, K. Sambasivaiah, R. Saxena; **Italy** E. Bajetta, A. Buonadonna, R. Buzzoni, R. Cannizzaro, A. Colao, C. De Angelis, P. Tomassetti; **Poland** J. Ćwikła, B. Kos-Kudła; **Slovakia** T. Salek; **Spain** J. Capdevila, G. Soler, J.M. Tabernero; **Sweden** H. Ahlman, M. Kjellman; **UK** G. Aithal, A. Anthoney, M. Caplin, A. Grossman, J. Newell-Price, J. Ramage, N. Reed, A. Rees, W. Steward, L. Wall; **USA** M. Choti, A.T. Phan, E.M. Wolin.

**Appendix 2**

**List of Ethics Committees and/or Institutional Review Boards**

| **Country/ Sitenumber** | **Ethics Committee** |
| --- | --- |
| Austria | Ethik-Kommission des Allgemeinen Krankenhauses und der Medizinischen Universität Wien Borschkegasse 8b/E06 1090 Wien |
| Belgium  056001 | Ethisch Comité, Universitair Ziekenhuis, De Pintelaan 185, 9000 Gent, Belgium |
| Belgium  056002 | Commission d'éthique biomédicale Hospitalo- Facultaire, Avenue Hippocrate 55.14, Tour Harvey - Niveau 0, 1200 Bruxelles, Belgium |
| Belgium  056003 | Ethisch comité UZA Wilrijkstraat 10 2650 Edegem Belgium |
| Czech Rep  203001 | Etická komise Vseobecne fakultni nemocnice v Praze Na Bojisti 1, 12808 Praha |
| Czech Rep  203002 | Eticka komise Fakultni nemocnice Na Bulovce Budinova 2, 180 00 Praha |
| Czech Rep  203003 | Etická komise FN a LF, UP Olomouc, I. P. Pavlova 6, 775 20 Olomouc |
| Denmark | Regional Ethics Review Board in Region Midtjylland, Skottenborg 26, 8800 Viborg |
| France  250 | C.P.P. ILE DE FRANCE XI 20, rue Armaglis 78105 SAINT-GERMAIN EN LAYE CEDEX |
| Germany | Landesamt für Gesundheit und Soziales Ethik-Kommission des Landes Berlin Fehrbelliner Platz 1 10707 Berlin |
| Greece  300001 | National Ethics Committee, Mesogion 284, Cholargos, 155 62 |
| India  356001 | Human Ethics Committee, Tata Memorial Centre, Dr. E. Borges Marg, Parel, Mumbai 400012, Maharashtra, INDIA |
| India 356002 | Institutional Ethics Committee,, Global Hospitals 6-1-1070/1 to 4 Lakidi ka pool Hyderabad 500004 State: Andhra Pradesh India |
| Italy  380001 | Fondazione IRCCS, Istituto Nazionale per lo Studio e la Cura dei Tumori, Via G Venezian 1, Milano 20133 |
| Italy  380002 | Azienda Sanitaria Ospedaliera ‘San Giovanni Battista de Torino’ C.so Bramanie 88/90 Torino 10126 |
| Italy  380003 | Centro di Riferimento Oncologico, Istituto Nazionale dei Tumori, Via Pedemontana Occidentale 12, Aviano 33081 |
| Italy  380004 | A.O.U.P. S. Orsola- Malpighi di Bologna, Via Albertoni 15, Bologna 40138 |
| Italy  380005 | A.O.U. Pisana Via Roma 67, Pisa 56126 |
| Italy  380007 | Centro di Riferimento Oncologico, Istituto Nazionale dei Tumori, Via Pedemontana Occidentale 12, Aviano 33081 |
| Italy  380008 | Universita degli Studi Ferderico II di Napoli, via Pansini 5, Napoli 80131 |
| Netherlands  528001 | Medisch Ethische Toetsings Commissie Erasmus MC DrMolewaterplein40 3015 GD Rotterdam Netherlands |
| Netherlands  528002 | Medisch Ethische Toetsings Commissie Universitair Medisch Centrum Groningen De Brug kamer 07.067 9700 RB Groningen Netherlands |
| Netherlands  528003 | Medisch Ethische Toetsings Commissie Universitair Medisch Centrum Utrecht Heidelbergglaan 100 Postbus 85500 3508 GA Utrecht Netherlands |
| Poland  CEC | Slaskiego Uniwersytetu Medycznego w Katowicach, ul. Poniatowskiego 15, Katowice 40-055 |
| Slovakia  703001 | Vychodoslovensky Onkologicky ustav a.s., P.O. Box D-47, Rastislavova 43, Kosice 04191 |
| Slovakia  703002 | Eticka komisia, Narodny onkologicky ustav, Klenova 1, 833 10 Bratislava, Slovakia |
| Spain  724001 | CEC: Comité Ético de Investigación Clínica of Hospital Universitari Vall d'Hebron, Institut de Recerca 3ª Planta Passeig de la Vall d'Hebron 119-129 08035 Barcelona Spain |
| Spain  724002 | CEC of site 724001 and the following LEC: CEIC Hospital Universitario de Bellvitge, Edificio Unitat de Recerca, Feixa Llarga, s/n 08907 L’Hospitalet de Llobregat, Barcelona, Spain |
| Spain  724005 | CEC of site 724001 and the following LEC: CEIC A1, Hospital General Universitario Gregorio Marañón, Pabellón de Gobierno, Dr. Esquerdo 46 28007 Madrid, Spain |
| Spain  724006 | CEC of site 724001 and the following LEC: CEIC, Hospital Universitario La Paz Paseo de la Castellana, 261 Escuela de Enfermería Planta 4ª Despacho 424 28046 Madrid, Spain |
| Spain  724007 | CEC of site 724001 and the following LEC: Comité Ético de Investigación Clínica Hospital Universitario Nuestra Sra. De Candelaria, Unidad de Calidad (3ª planta de la Residencia General, nº 331) Carretera del Rosario, 145, 38010 Santa Cruz de Tenerife, Spain |
| Sweden | Regional Ethical Review Board in Uppsala,Drottninggatan 4, 753 09 Uppsala |
| UK | NRES Committee London-South East, South East Coast Strategic Health Authority, Preston Hall, Aylesford, Kent ME20 7NJ |
| UK – Change of Ethics Committee | NRES Committee London – South East, Room 4W/10, 4^th^ Floor West, Charing Cross Hospital, Fulham Palace Road, London W6 8RF |
| USA  840001 | Providence Portland Medical Center Institutional Review Board, 5251 NE Glisan St., Bldg A, 3rd Floor Portland, OR 97213 |
| USA  840002 | Western Institutional Review Board (WIRB) 3535 Seventh Ave. SW PO Box 12029 Olympia, WA 98502- 20209 |
| USA  840003 | The University of Texas M.D. Anderson Cancer Center Institutional Review Board, 1515 Holcombe Blvd., Unit 1437, Houston, TX 77030 |
| USA  840004 | Johns Hopkins, Medicine, IRB, Reed Hall B-130, 1620 McElderry St. Baltimore, MD 21205- 1911 |
| USA  840005 | Cedars-Sinai Medical Center, Office of Research Compliance, 8383 Wilshire Blvd., Suite 742, Beverly Hills, CA, 90211 |
| USA  840007 | Roger Wilson, MD Chairman, Institutional Review Board, Memorial Sloan-  Kettering Cancer  Center, 1275 York Avenue New York, NY 10065 |
| USA  840009 | Institutional Review, Board, Dana-Farber Cancer, Institute, Dana-Farber/Harvard, Cancer Center, 44 Binney Street, OS229, Boston, MA 02115 |
| USA  840010 | OHSU Regulatory, Integrity Office, (ORIO), 3181 SW Sam Jackson, Park Road, Portland, OR 97239 |
